# MGMT promoter methylation status and MGMT and CD133 immunohistochemical expression as prognostic markers in glioblastoma patients treated with temozolomide plus radiotherapy

**DOI:** 10.1186/1479-5876-10-250

**Published:** 2012-12-17

**Authors:** Consolación Melguizo, Jose Prados, Beatriz González, Raul Ortiz, Angel Concha, Pablo Juan Alvarez, Roberto Madeddu, Gloria Perazzoli, Jaime Antonio Oliver, Rodrigo López, Fernando Rodríguez-Serrano, Antonia Aránega

**Affiliations:** 1 Institute of Biopathology and Regenerative Medicine (IBIMER), Granada 18100, Spain; 2 Department of Anatomy and Embriology, University of Granada, Granada 18012, Spain; 3 Service of Medical Oncology, Virgen de las Nieves Hospital, Granada, 18014, Spain; 4 Department of Health Science, University of Jaén, Jaén, 23071, Spain; 5 Anatomopathological Service, Virgen de las Nieves Hospital, Granada, 18014, Spain; 6 Departament of Biomedical Science - Histology, University of Sassari, Sassari, Italy; 7 National Institute of Biostructures and Biosystems, INBB, Sassari, Italy

**Keywords:** Glioblastoma, Radiotherapy, Temozolomide, MGMT, Methylation, CD133

## Abstract

**Background:**

The CD133 antigen is a marker of radio- and chemo-resistant stem cell populations in glioblastoma (GBM). The O^6^-methylguanine DNA methyltransferase (MGMT) enzyme is related with temozolomide (TMZ) resistance. Our propose is to analyze the prognostic significance of the CD133 antigen and promoter methylation and protein expression of MGMT in a homogenous group of GBM patients uniformly treated with radiotherapy and TMZ. The possible connection between these GBM markers was also investigated.

**Methods:**

Seventy-eight patients with GBM treated with radiotherapy combined with concomitant and adjuvant TMZ were analyzed for MGMT and CD133. MGMT gene promoter methylation was determined by methylation-specific polymerase chain reaction after bisulfite treatment. MGMT and CD133 expression was assessed immunohistochemically using an automatic quantification system. Overall and progression-free survival was calculated according to the Kaplan–Meier method.

**Results:**

The MGMT gene promoter was found to be methylated in 34 patients (44.7%) and unmethylated in 42 patients (55.3%). A significant correlation was observed between MGMT promoter methylation and patients’ survival. Among the unmethylated tumors, 52.4% showed low expression of MGMT and 47.6% showed high-expression. Among methylated tumors, 58.8% showed low-expression of MGMT and 41.2% showed high-expression. No correlation was found between MGMT promoter methylation and MGMT expression, or MGMT expression and survival. In contrast with recent results, CD133 expression was not a predictive marker in GBM patients. Analyses of possible correlation between CD133 expression and MGMT protein expression or MGMT promoter methylation were negative.

**Conclusions:**

Our results support the hypothesis that MGMT promoter methylation status but not MGMT expression may be a predictive biomarker in the treatment of patients with GBM. In addition, CD133 should not be used for prognostic evaluation of these patients. Future studies will be necessary to determine its clinical utility.

## Background

Glioblastoma (GBM), the most common primary brain tumor in adults, is a rapidly progressive and fatal disease with a low median overall survival [1]. The treatment of these tumors with temozolomide (TMZ) introduces alkyl groups into DNA preventing its replication.This structural modification induces cell death. However, DNA-repair proteins, such as O^6^-alkylguanine DNA alkyltransferase (AGT), are able to remove alkyl adducts from the O^6^ position of guanine. Which is especially harmful, and the O^4^ position of thymine, restoring these DNA bases and preventing TMZ-induced cell death [2]. The DNA-repair protein AGT is encoded by the gene O^6^-Methlyguanine-DNA-methyltransferase (MGMT). Determination of promoter methylation of the MGMT gene is being included as a relevant factor of the patient molecular profile [3]. Although epigenetic silencing of the MGMT gene promoter has been associated with prolonged survival in glioblastoma patients [4], there is much controversy about its use as a prognostic marker for the response of patients with newly diagnosed glioblastoma to temozolomide [5,6]. Moreover, whether any correlation exists between MGMT protein expression and promoter hypermethylation and patient outcomes has not been elucidated. Therefore, various studies using different assessments have reported different results [7-11]. These inconsistencies may be caused by intratumoral heterogeneity, different evaluation methods, and different cut-off values.

The cancer stem cell (CSC) theory postulates that tumors arise from a subpopulation of cells that are characterized by self-renewal, infinite proliferative potential, multipotency, and their ability to initiate new tumors in vivo [12]. Interestingly, CSC cells are postulated to be mediators of radio- and chemo-resistance. Tumor cells with stem-like features have been identified in glioblastoma [13]. These cells express the transmembrane glycoprotein prominin-1 (CD133) (a cell-surface marker expressed on normal human neuronal stem cell) and have the ability to initiate a tumor in vivo after xenotransplantation in mice. Few data are available on the actual prognostic impact of CD133 expression in malignant gliomas. Glioblastoma stem cells are highly resistant to conventional chemotherapy and radiotherapy [14,15] and the chemo-radioresistance of these cells may be responsible for the poor clinical outcome of these patients.

The aim of our study is to determine the prognostic significance of MGMT by analyzing both MGMT gene promoter methylation and protein expression in a homogenous series of GBM patients treated with radiotherapy and temozolomide. In addition, we evaluated the immunohistochemical expression of CD133 investigating its association with MGMT and clinical outcomes.

## Methods

### Tissue samples

Samples were obtained from the Anatomopathological Service of Hospital Virgen de las Nieves from Granada (Spain) and the University Hospital of Sassari (Italy), from 2001 to 2009. The Ethics Committees of both Hospitals approved the collection and use of human brain tumor tissue samples. We obtained tumor tissue samples from 78 patients with newly diagnosed GBM which was histologically confirmed and Karnofsky performance score (KPS) ≥ 60. Patients were selected regardless of extent of surgery. All patients had been treated with concurrent chemo-radiotherapy (2 Gy per fraction, once a day, five days a week, 60 Gy total dose) with concomitant TMZ (75 mg per square meter of body surface area per day for seven days a week from the first to the last day of radiotherapy) followed by adjuvant TMZ (200 mg per square meter of body surface area on days 1 to 5 given at four-weekly intervals). The patient characteristics are summarized in Table 1.

**Table 1:** Patient characteristics (n = 78)

Age (years)	Mean	56
	Range	24–81
Gender	Male	42 (53.8%)
	Female	36 (46.1%)
Tumor location	Frontal	20 (25.6%)
	Parietal	13 (16.6%)
	Temporal	13 (16.6%)
	Occipital	11 (14.1%)
	More than one lobe	21 (26.9%)
Duration of symptoms prior to diagnosis	< 3 months	60 (76.9%)
	≥ 3 months	18 (23.1%)
Karnofsky performance score	≥ 60	78 (100%)

Patients age 70 years or older with newly diagnosed GBM and postoperative Karnofsky performance score (KPS) greater than 60 were eligible for this nonrandomized phase II trial

### DNA extraction, bisulfite treatment and methylation-specific PCR

DNA was extracted according to standard protocols. Methylation patterns in the CpG island of MGMT were determined by chemical modification of unmethylated, but not methylated, cytosine to uracil. Methylation-specific PCR (MSP) was performed with primers specific for either modified-methylated or unmethylated DNA, as described previously [16]. DNA (2 μg) was denatured with sodium hydroxide and modified with sodium bisulfite. DNA samples were then purified (EpiTect Bisulfite Conversion). Primer sequences for the unmethylated reaction were 5^′^-TTTGTGTTTTGATGTTTGTAGGTTTTTGT-3^′^ (forward primer) and 5^′^-AACTCCACACTCTTCCAAAAACAAAACA-3^′^ (reverse primer), and for the methylated reaction, they were 5^′^-TTTCGACGTTCTAGGTTTTCGC-3^′^ (forward primer) and 5^′^-GCACTCTTCCGAAAACGAAACG-3^′^ (reverse primer). Amplified products of PCR were electrophoresed on 3% agarose gels, were visualized by staining with ethidium bromide, and were examined under UV illumination.

### Immunohistochemistry

Antibodies for MGMT (1:50; Santa Cruz Biotechnology, Inc.) and CD133 (1:50, Abcam, Cambridge, UK) were used for immunohistochemical analysis. Immunostaining was performed using the Bond Polymer Refine Detection system (Leica Microsistemas S.L.U, Barcelona, Spain). Briefly, representative paraffin blocks were cut consecutively at a thickness of 4 mm, and immunohistochemical staining was carried out using the Microprobe Immuno/DNA stainer (Fisher Scientific, Tustin, CA, USA). Sections were deparaffinized in xylene and treated with 0.3% hydrogen peroxide in methanol for 20 min to block endogenous peroxidase activity. The sections were washed in phosphate-buffered saline and then incubated with primary antibodies for 60 min. The samples were then incubated in secondary antibody for 8 min. The substrate chromogen, 3.3^′^-diaminobenzidine (DAB), enabled visualization of the complex via a brown precipitate. Hematoxylin (blue) counterstaining enabled the visualization of the cell nuclei. Omission of primary antibody served as a negative control. Readings were taken automatically with the ACIS III DAKO system for quantification immunohistochemistry and were verified by two experienced pathologist. The percentage of stained tumor cells was scored as +/− (<10%), 1+ (10% to 25%), 2+ (25% to 50%), 3+ (>50%). For statistical analysis, scores of +/− and 1+ were defined as low-expression group and scores of 2+ and 3+ were defined as high-expression.

### Statistical analysis

Overall survival (OS) was calculated from the date of the diagnosis. The progression-free survival (PFS) was calculated from the date of the progression, according of MacDonald criteria [17]; size and volume ≥ 25% of initial measurements, or appearance of a new lesion, or if the patient’s neurologic condition worsened and required an increased dose of steroids. The PFS and OS curves were estimated by the Kaplan-Meier method and compared using the two-sided log-rank test. A multivariable analysis was done using the Cox proportional hazards regression to determine the prognostic effect of variables. Contingency tables were analyzed by *X*^2^ and Fisher’s exact test. The McNemar test was applied to compare variables before and after treatment. All calculations were made using the statistical software SPSS, version 15.0. Statistical significance was set at the level of P < 0.05.

## Results

### MGMT promoter methylation status and MGMT protein expression

**Figure 1:**
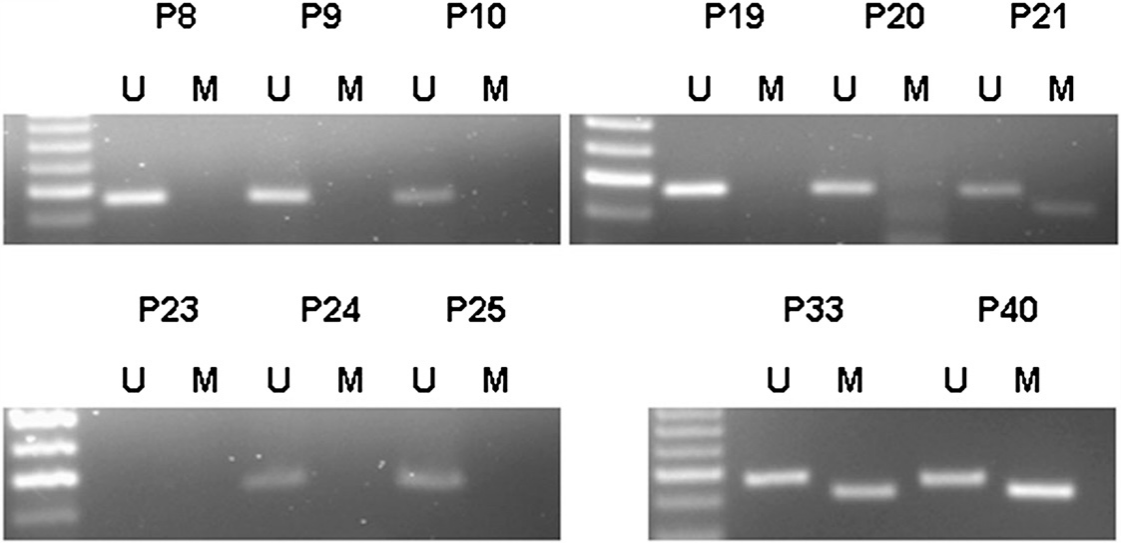
**Representative methylation-specific PCR (MSP) analyses of the MGMT promoter in GBM tissue from eleven patients (P).** Note the presence of bands in both the unmethylated (U, 93 bp) and methylated (M, 81 bp) lanes for glioblastoma samples 21, 33 and 40, reflecting a methylated MGMT promoter. The lack of a band in the lane corresponding to methylation-specific primers for GBM in the rest of the samples reflects the absence of MGMT promoter methylation. In some samples, such as sample 23, no PCR products were observed (sample excluded).

**Figure 2:**
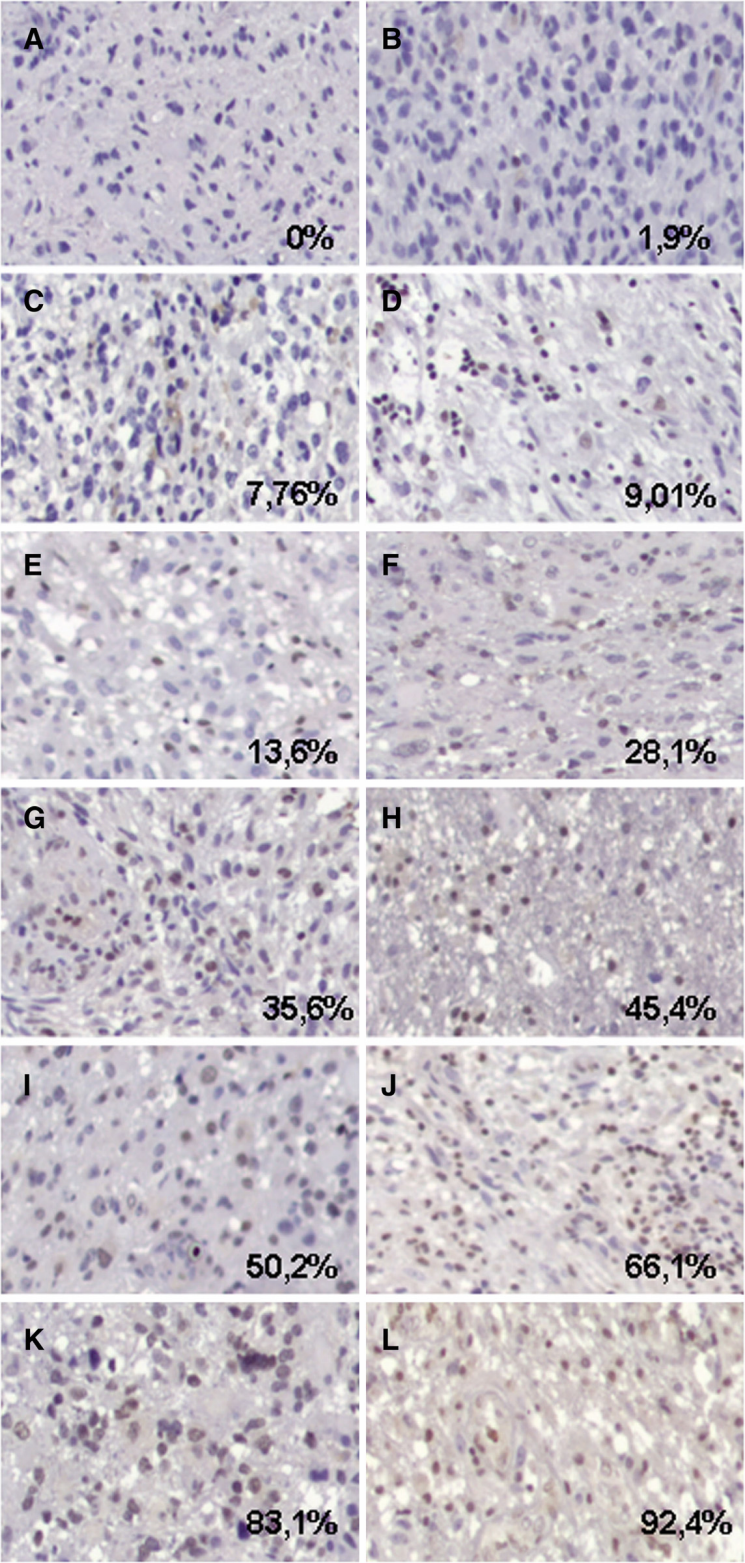
Representative photomicrographs illustrating different percentages of MGMT-stained tumor cells scored as +/− (A, B, C and D), 1+ (E), 2+ (F, G, H and I), 3+ (J, K and L) (see Methods) (20×).

The methylation status of the MGMT promoter and MGMT protein expression was determined for 76 of the 78 tumors (97.4%). Two of the 78 GBM cases were excluded due to unsuccessful PCR amplification. MGMT promoter methylation was detected in 44.7% (34/76) of the GBM samples analyzed by MSP (Figure 1). A positivity score of 1+, 2+ and 3+ were detected in 31 (40.8%), 19 (25%) and 15 (19.7%) cases respectively (Figure 2). Only 11 (14.5%) cases showed a score of +/− (Table 2). For the correlation of MGMT promoter methylation with MGMT protein expression, the scores +/− and 1+ were categorized into a low-expression group (42, 55.3%), and scores 2+ and 3+ into a high-expression group (34, 44.7%). Among the 42 unmethylated tumors, 22 (52.4%) showed low-expression of MGMT and 20 (47.6%) showed high-expression while among the 34 methylated tumors, 20 (58.8%) showed low-expression of MGMT and 14 (41.2%) showed high-expression. No correlation between MGMT protein expression and MGMT promoter methylation was observed (P = 0.903).

**Table 2:** Association of MGMT promoter methylation and MGMT and CD133 expression in human GBM

		**Total**	**Methylated**	**Unmethylated**	**Not done**
**MGMT expression**					
Low-expression	+/−, +	42 (55.3%)	20 (26.3%)	22 (29%)	2 (2.6%)
High-expression	++, +++	34 (44.7%)	14 (18.4%)	20 (26.3%)	
**CD133 expression**					
Low-expression	+/−, +	41 (54.7%)	16 (21.3%)	25 (33.4%)	3 (4%)
High-expression	++, +++	34 (45.3%)	18 (24%)	16 (21.3%)	

Data are represented as the percentage of the total of analyzed patients.

### CD133 protein expression

**Figure 3:**
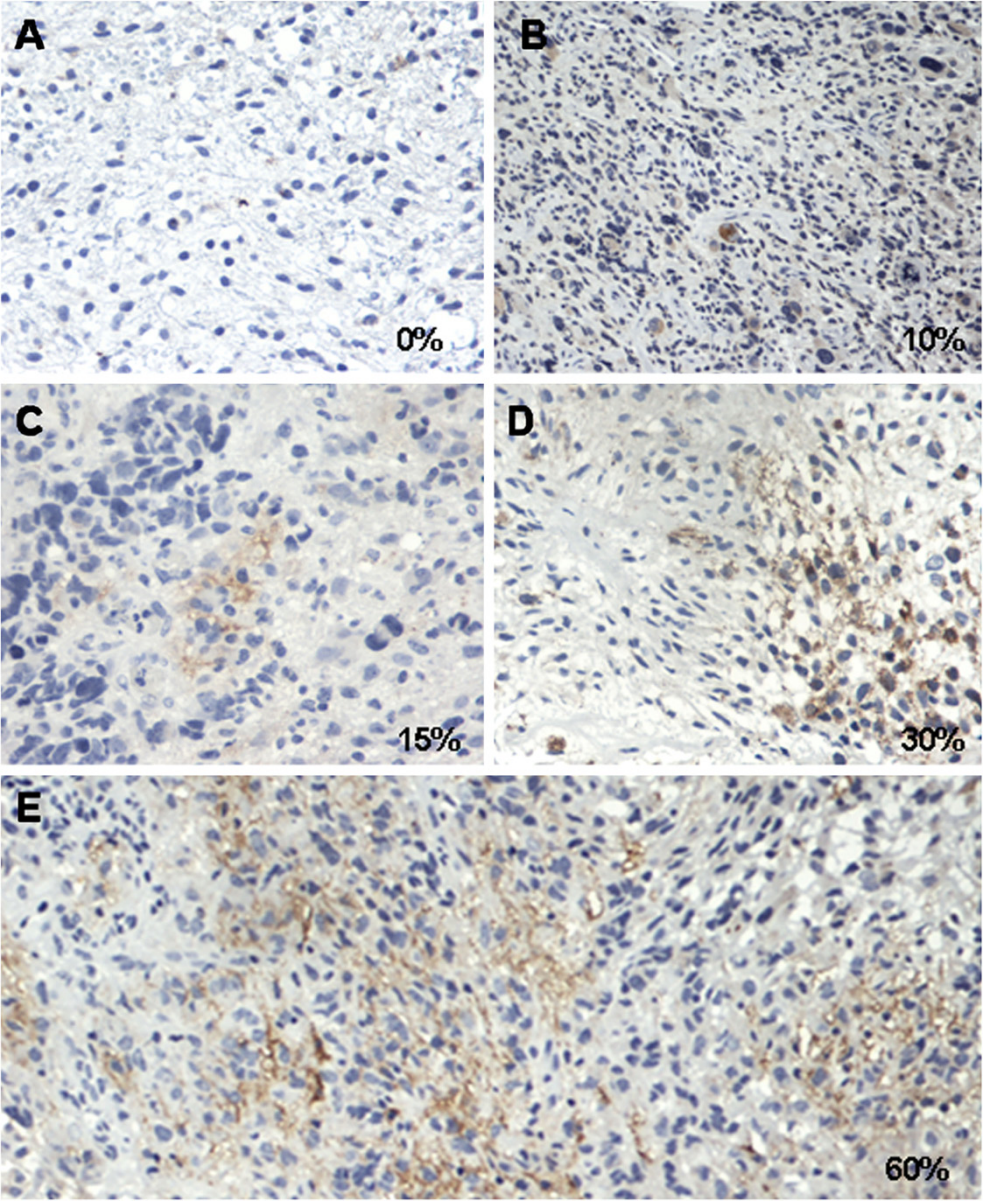
Representative photomicrographs illustrating different percentages of CD133 stained tumor cells corresponding to being scored as +/− (A), 1+ (B and C), 2+ (D), 3+ (E) (see methods) (20×).

CD133 expression was available for 75 of the 78 patients (96.2%). Among the 75 samples, 26 (34.7%), 27 (36%) and 7 (9.3%) were included as scores 1+, 2+ and 3+ respectively. Fifteen (20%) cases showed a score of +/− (Figure 3). As was done previously, to correlate MGMT and CD133 protein expression, the scores were categorized into a low-expression group (41, 54.7%), and a high-expression group (34, 45.3%) (Table 2). Analysis of a possible correlation between CD133 expression and MGMT protein expression or MGMT promoter methylation was negative.

### Influence of MGMT promoter methylation status and MGMT and CD133 protein expression on overall survival

**Figure 4:**
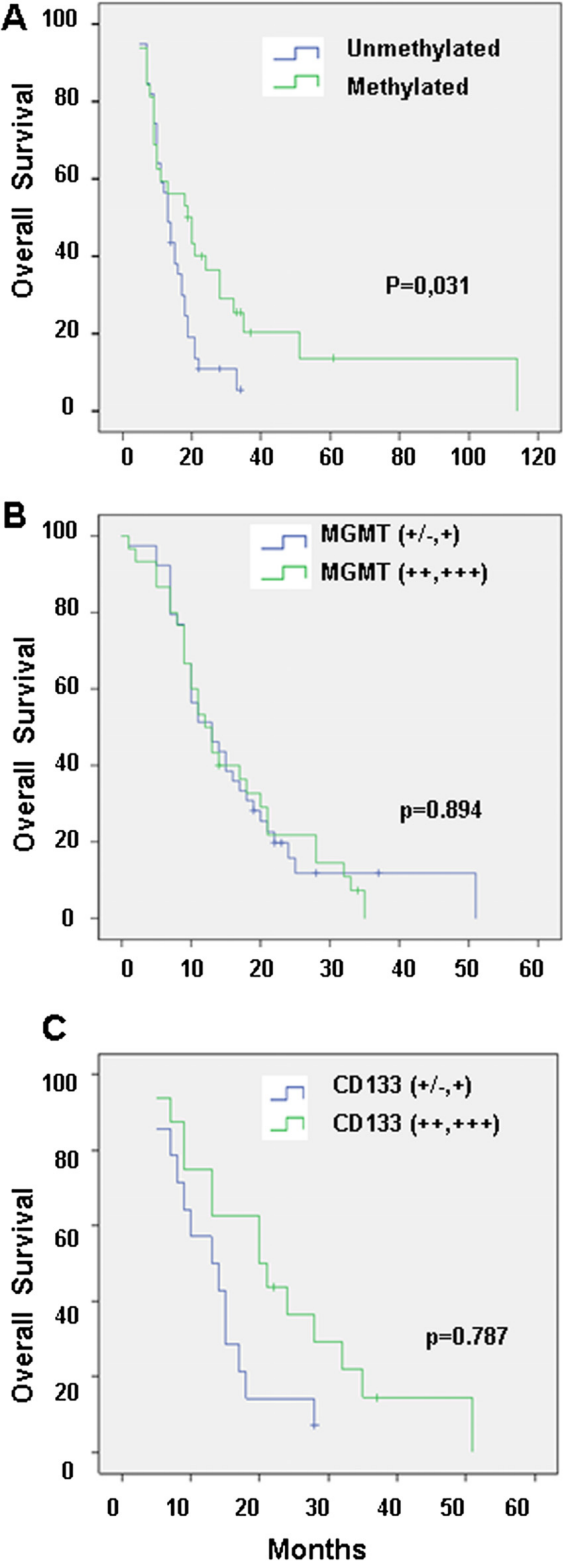
Overall survival curves of patients with GBM according to MGMT methylation status (A), MGMT expression (B) and CD133 expression (C).

Statistical analysis showed a significant correlation between OS and MGMT promoter methylation status (Figure 4A). The median OS among patients with methylated MGMT promoter tumors was 19 months (95% CI, 9.6–28.4 months) compared with 13 months (95% CI, 10.5–15.4 months) in patients with unmethylated MGMT promoter tumors (log-rank, P = 0.031). In contrast, OS showed no statistically significant differences when it was correlated with MGMT expression (log-rank, P = 0.894) (Figure 4B). The median OS among patients with high-expression of MGMT was 12 months (95% CI, 8.8–15.2 months) compared with 13 months (95% CI, 8.1–17.9 months) in low-expression of MGMT tumors (log-rank, P = 0.894). In addition, no differences were observed between survival estimates of patients with CD133 low-expression tumors and CD133 high-expression tumors (Figure 4C). The median OS among patients with CD133 high-expression tumors was 14 months (95% CI, 9.1–18.9 months) compared with 13 months (95% CI, 6.1–19.8 months) in CD133 low-expression tumors (log-rank, P = 0.787).

### Influence of MGMT promoter methylation status and MGMT and CD133 protein expression on progression-free survival

**Figure 5:**
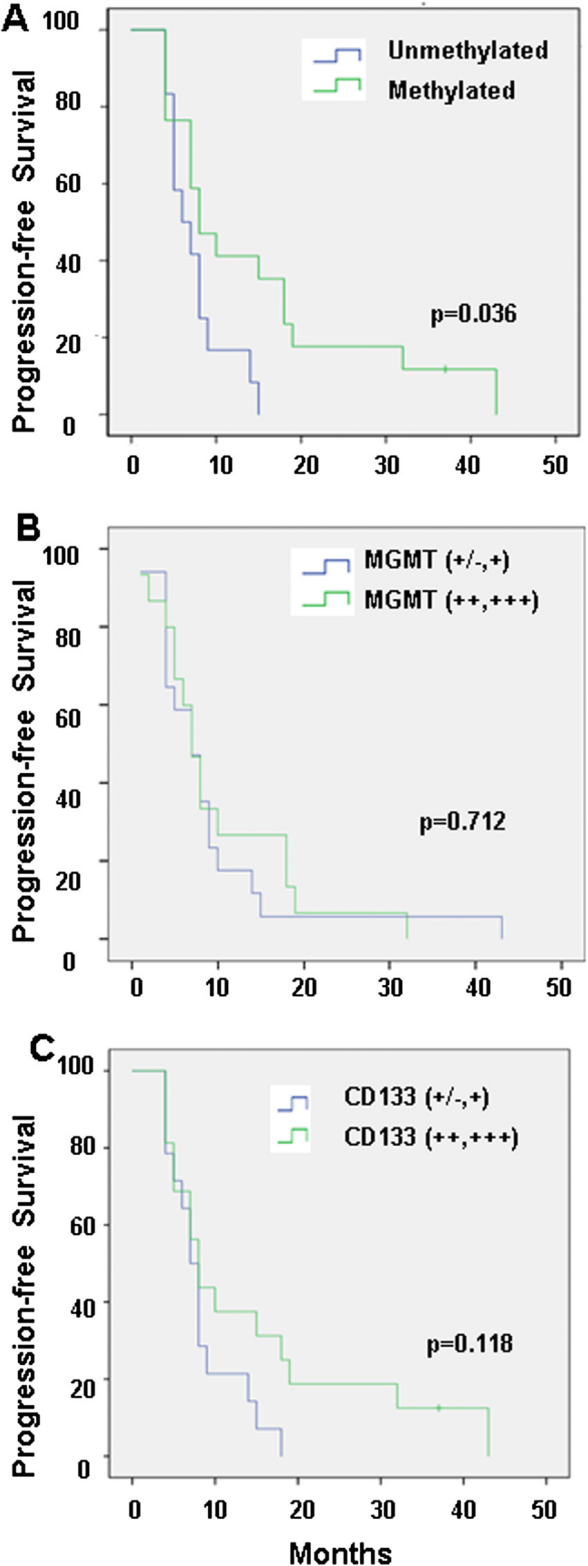
Progression-free survival curves of patients with GBM according to MGMT methylation status (A), MGMT expression (B) and CD133 expression (C).

The median PFS was 8 months (95% CI, 4–12 months) for the methylated MGMT promoter status compared with 6 months (95% CI, 2.6–9.4 months) for the unmethylated MGMT promoter status, showing a significant correlation (log-rank, P = 0.036) (Figure 5A). By contrast, no significant correlation was observed between PFS and MGMT protein expression (Figure 5B). Finally, in these groups, the median PFS was 7 months (95% CI, 5.2–8.8 months) for low-CD133 expression tumors compared with 8 months (95% CI, 6.7–9.3 months) for high-CD133 expression tumors (P = 0.118) (Figure 5C).

### Influence of MGMT promoter methylation status and MGMT and CD133 protein expression on treatment response

All patients were assessed for clinical and radiological response. The statistical analysis showed that only the MGMT promoter status significantly correlated with radiological response (P = 0.036). The response rate was significantly higher among patients with a methylated MGMT promoter status versus an unmethylated status (43.3% and 17.5%, respectively) (P = 0.036) (Table 3).

**Table 3:** **Radiologic response of GMB patients following Macdonald et al.**[17]

	**Radiologic response**	
**MGMT**	**Negative**	**Positive**
Unmethylated	33 (82.5%)	7 (17.5%)
Methylated	17 (56.7%)	13 (43.3%)
Total	50 (71.4%)	20 (28.6%)

The methylation of MGMT showed a significant correlation with radiologic response (p < 0.05).

### MGMT promoter methylation and MGMT and CD133 protein expression on recurrent GBM

This study included 11 patients of the 78 patients who underwent reoperation for tumor recurrence after radio-chemotherapy. One of the 11 GBM cases was excluded. In spite of the few samples, there were no significant differences between variables before and after treatment with radiotherapy and temozolomide (McNemar test) (Table 4).

**Table 4:** Modulation of MGMT promoter methylation

**Patient**	**Pre-treatment**			**Post-treatment**		
	**MGMT**	**MGMT**	**CD133**	**MGMT**	**MGMT**	**CD133**
	**Promoter**			**Promoter**		
**Patient 1**	M	+	++	M	+/−	+
**Patient 2**	M	++	+	U	++	+/−
**Patient 3**	U	+++	+	U	+	++
**Patient 4**	M	++	+	M	++	+
**Patient 5**	M	+	++	U	+	+
**Patient 6**	U	+	+	M	+	+
**Patient 7**	U	+	+	U	+	+++
**Patient 8**	M	+	++	U	+	++
**Patient 9**	U	+/−	+	-		-
**Patient 10**	U	+/−	+	U	++	++
**Patient 11**	M	++	+	M	+++	+

MGMT and CD133 in recurrent GBM before and after treatment with radiotherapy and temozolomide.

## Discussion

Clinical trials have demonstrated a significantly prolonged median survival of GBM patients treated with TMZ associated to radiotherapy after surgical resection [18-20]. In addition, a strong correlation between the MGMT gene promoter methylation status and the TMZ treatment effect and outcome was shown by Hegi et al. [4], confirming previous results which correlated MGMT inactivation and clinical response to alkylating agents [16,21]. Criniere et al. [22] showed that while the MGMT promoter methylation status had no impact on the OS of GBM patients treated with alkylating agents, it did have an impact on those treated with chemo-radiotherapy, suggesting that the prognostic impact of this methylation is dependent on therapeutic modalities. In fact, Weller et al. [23] analyzed patients with GBM treated with radiotherapy, with chemotherapy and with both radiotherapy and chemotherapy and showed that the methylation status was a significant prognostic factor for OS and PFS only in the subgroup of patients treated with radiotherapy and concurrent TMZ. These results have been recently confirmed by Cao et al. [8], Minniti et al. [24] and Kim et al. [25]. Recently, the methylation status was analyzed in elderly patients with GBM and was found to be strictly correlated with the pattern of, and time until, GBM recurrence [26], but not with its evolution after recurrence [27]. Rivera et al. [28] demonstrated that MGMT promoter methylation was a predictor of survival in GBM treated exclusively with radiotherapy. In addition, a randomized phase III trial comparing standard adjuvant TMZ with a dose-dense schedule in newly diagnosed GBM confirmed the prognostic significance of MGMT methylation in GBM [29]. However, previous studies considered promoter methylation of the MGMT gene not to be a reliable prognostic factor of responsiveness to alkylating agents in glioblastomas [30]. Recent studies have also questioned the role of promoter methylation status of MGMT in GBM. Yachi et al. [31] failed to establish this correlation in a larger number of patients and showed that neither MSP-MGMT methylation nor immunohistochemical MGMT expression had prognostic implications in GBM patients. Similarly, Tang et al. [32] did not find a correlation between progression-free survival and MGMT promoter methylation in chinese patients. In this context, we conducted a retrospective study in a homogeneous series of patients diagnosed with GBM treated with radiotherapy and concurrent temozolomide using MSP, which has been proposed as the most convenient technique in clinical routine diagnostics [33]. We found that the percentage of methylation of the MGMT promoter to be higher than that reported by Hegi et al. [4] but similar to those reported by other authors [22,34]. The MGMT methylation status was clearly confirmed as an independent prognostic factor of GBM PFS and OS. In addition, patients with a methylated MGMT promoter status had higher response rates to TMZ and radiation therapy compared to those with a non-methylated status, so that MGMT methylation was a predictive factor for radiologic response

On the other hand, the association between MGMT protein expression and promoter methylation in vivo has been widely discussed. Several studies have reported a significant association of high MGMT expression and poor prognosis of patients [7-10,35-38]. We analyzed the expression of MGMT by immunochemistry using a digital quantitative method to avoid observer variability [39]. Our study clearly showed no correlation between MGMT expression and MGMT promoter methylation, supporting the finding observed by Rodriguez et al. [40] and Uno et al. [41], or overall survival or radiological response coinciding with those findings of Preusser et al. [11]. The regulation of the MGMT gene is a complex phenomenon in which promoter hypermethylation is one of the factors that influence the final expression of the protein. In this context, the recent demonstration that there is discordance between MGMT promoter methylation and levels of MGMT mRNA expression suggests that other mechanisms may regulate the expression of this enzyme [42]. It also possibly suggests that promoter methylation and expression alone are not sufficient to provide information on the expected clinical course in patients with malignant glioma who receive chemotherapy with alkylating agents. In fact, a comprehensive study undertaken by sequencing the MGMT gene promoter has shown a strong correlation between the methylation site and treatment response [43].

CD133, a five-transmembrane cell surface protein found in human stem cells from various sources including the central nervous system [44], has been proposed to detect GBM CSCs. Considering the inherent resistance of CSCs to chemotherapy and radiotherapy [14,15], it has been hypothesized that the clinical outcome will be inversely related to the presence of CSC-marker-positive cells. However, the impact of the presence of CSC in the clinical progression of tumors is unknown. Few studies exploring the prognostic value of CD133 expression as a marker of CSC in GBM have been undertaken; most were heterogeneous and used different methods (QRT-PCR, immunohistochemistry, FACS) so that comparisons are difficult. Intense CD133 expression was detected in high-grade oligodendroglial tumors [45] and in grade II-IV gliomas [46], both with poor prognoses. Similar results were founded in grade IV gliomas where CD133 expression was related to OS and PFS [47]. Murat et al. [48] provided evidences that the glioblastoma stem cell phenotype (including CD133 expression) correlated with chemoradiotherapy resistance and patient survival. In a study with very homogeneous samples of GBM, high expression of CD133 was found to be an unfavorable prognostic factor [49]. In addition, an in vitro study using CSC obtained from 44 GBM patients to evaluate CD133/Ki67 expression by immunohistochemical analysis, concluded that CD133+ was correlated to survival [50]. However, the same authors demonstrated that high expression of CD133 was associated with a better prognosis when detected by FASCcan [51]. Similar results were previously found by Joo et al. [52] who described CD133 as a favourable prognostic factor for GBM. In this context, our study clearly demonstrates that CD133 has no implication in the prognoses of GBM patients supporting similar results of Kim et al. [53]. Finally, the prognostic impacts of variations of MGMT promoter methylation or MGMT and CD133 protein expression after treatment, are not known. A previous study determined that MGMT methylation status has no prognostic value after GBM recurrence [27]. Our preliminary study in samples obtained before and after treatment from only ten patients with recurrent GBM suggested that there are no differences between the analyzed variables although the low number of patients does not allow to obtain statistically significant conclusions.

## Conclusions

Our study was designed to analyze the correlations between clinical features, MGMT status (promoter methylation and gene expression) and CD133 expression, and outcome in a set of patients uniformly treated with concomitant and adjuvant TMZ and chemo radiation. In this homogeneous series, the prognostic significance of MGMT promoter methylation has been clearly demonstrated. However, MGMT protein expression showed no correlation with MGMT promoter methylation. In addition, CD133 expression was correlated neither with the survival of patients with GBM nor with MGMT. In light of our data, together with the controversies reported in the literature, further studies are warranted to clarify whether MGMT and CD133 can discriminate between biologically distinct groups of GBM.

## Abbreviations

AGT: O^6^-alkylguanine DNA alkyltransferase; CSC: Cancer stem cell; DAB: 3.3^′^-diaminobenzidine; GBM: Glioblastoma; MGMT: O^6^-Methlyguanine-DNA-methyltransferase; OS: Overall survival; PCR: Polymerase chain reaction; PFS: Progression-free survival; TMZ: Temozolamide.

## Competing interests

The authors declare that they have no competing interests.

## Authors’ contributions

CM, JP and AA participated in the project design, coordination the experiments, and manuscript preparation. RO, JAO and FRS carried out bisulfite treatment and methylation-specific PCR. AC, PJA and RL carried out immunohistochemical analysis. BG and RM participated in the collected and analyzed the clinical data. All authors read and approved the final manuscript.
